# Supplementary material for: Content-rich biological network constructed by mining PubMed abstracts
Source: BMC Bioinformatics. 2004 Oct 8;5:147. doi: 10.1186/1471-2105-5-147 (PMC528731; doi:10.1186/1471-2105-5-147)
Supplement: Additional File 5 — The original Chilibot query results of the term "long-term potentiation (LTP)" and 22 other terms, limiting the latest references analyzed to the years 1990, 1995, 2000, and 2004. [file 1471-2105-5-147-S5.bz2 › chilibotAdditionalFile5/ltp2004/html/ZIF268_CAMKII.html]

 


 **ZIF268** and **CAMKII** 
  
Found 2 abstracts in PubMed,  **2 abstracts were retrieved and analyzed**.  


---

 Search Google  |
 PDF files only 
|  EDU domain only 

---

**Interactive relationship** (e.g. stimulation, inhibition, etc)

- Co transfection with  **CaMKII**  stimulated nuclear translocation of  **Zif268** .  Ref: 12421350 J Neurochem, 2002
- Increased activity by  **Zif268**  was additive with  **CaMKII**  induced activation but not with activation induced by MEKK.  Ref: 12421350 J Neurochem, 2002
- **Zif268**  is a potential transcription factor for the  **CaMKII**  dependent pathway.  Ref: 12421350 J Neurochem, 2002
